# Diet and Hygiene in the Treatment of Diabetes

**Published:** 1887-06

**Authors:** 


### DIET AND HYGIENE IN THE TREATMENT OF DIABETES.

PROFESSOR C. W. PURDY advises a rigid enforcement of the following diet and hygiene in cases of confirmed diabetes mellitus, in which the matter of diet and hygiene is of more importance than medication.

What to Eat.—Fresh meats of all kinds. Meats, salted, smoked, cured, potted, or preserved in any way except with honey or sugar. Poultry and game of every kind. Eggs in every shape. Bread and biscuits made with prepared gluten bran, or almond flour. Fish of every kind, fresh and preserved, except oysters. Soups of all kinds made without flour or prohibited vegetables. Butter, cheese, cream, curds, oil, dripping and suet, green vegetables, summer cabbage, cauliflower, spinach, broccoli tops, Brussell's sprouts, turnip tops, string beans, watercresses, mustard and cress, sauer kraut, the green parts of lettuce, sorrel, mushrooms, celery tops, turnip tops, cucumbers, pickles, olives, young onion tops. Nuts of various kinds (except chestnuts), almonds, filberts, walnuts, Brazil nuts, cocoanuts, butternuts, pecans, etc. Condiments in moderation.

What to Drink.—Water, soda, seltzer and all mineral waters, Tea and coffee without milk or sugar. Unsweetened spirits, as cognac, whisky, gin, rum, etc., claret, red burgundy, the driest pale sherry, Amontillado or Vino de Pasto.

What to Avoid.—Milk, except very small quantities for cooking purposes, skimmed milk or whey. The liver of all animals. Oysters, the interior of crabs, lobsters, etc. Common flour, biscuits, rusks, toast. Farinaceous vegetables—as potatoes, rice, oatmeal, corn flour, sago, tapioca, arrowroot, maccarroni and vermicelli, barley meal, etc. Saccharine vegetables—turnips, carrots, parsnips, peas, French beans, beets, onions, Jerusalem artichokes, asparagus, rhubarb, tomatoes. Blanched vegetables of all kinds—celery, sea kale, endive, radishes; also stalks and white parts of such vegetables as cabbage, lettuce, broccoli, etc. Fruits of all kinds, jams, syrups, sugars, honey. Certain condiments, as chutnee and sweet pickles, cocoa and chocolate. All malt liquors, with exceptions specified; cider, champagne and all sparkling wines; port, Madeira and all sweet wines; sherry, except the driest; sweetened spirits and liqueurs.

Special Suggestions.—Price's glycerine may be used as a substitute for sugar and syrup. An egg beaten up in tea or coffee forms a substitute for milk. The most palatable form of prepared bread is the gluten roll; this cut into slices, well buttered, may be advantageously eaten with potted meats, anchovy paste, caviar, etc.

An agreeable omelette may be made with four eggs to one tablespoonful of milk.

Melted butter without flour, may be prepared as follows: Rub up the yolk of a hard egg-boiled with olive oil until a rich paste is formed, add two ounces of butter, and heat, gently stirring; add anchovy sauce, capers, fennel, chopped parsley or sorrel, lemon juice, etc., as may be required.

A green salad may be served with meat; it should be mixed with plain oil or vinegar, but the cook should be warned against adding sugar thereto.

As the number of green vegetables permitted is limited, they should be fresh, carefully cooked, and well served. Watercresses should be served at every meal. If the patient will accustom himself to the use of sauer kraut regularly, it will be beneficial.

Meat twice cooked should never be used. Since sweets may not be partaken of, savories should follow meats. Savory omelettes, caviar, anchovies, cheese, fondûs, etc. Sometimes the omelettes may be flavored with grated cocoanut, glycerole of orange peel, essence of vanilla, almonds, etc., or a mixture of sherry (dry), glycerine and lemon juice.

The patient should avoid too great indulgence of his appetite; he should just satisfy his hunger and no more. After a little restraint the craving for extra meat soon ceases.

A list of dishes should be drawn up appropriate for each meal, and these should be as varied as possible.

The patient should carry out scrupulously the diet list above recommended, remembering that a well-regulated diet influences his disease more powerfully for good than does medication. If he grows impatient of the restraint, he should remember that many races live in perfect health upon a far more limited diet, thus: dried flesh or pemmican suffices to keep the Indian or trapper in full vigor for unlimited periods.

Hygiene.—The freshest and purest of air should be breathed; hence the apartments must be perfectly ventilated. Especially must exposure to irrespirable gases be avoided. The patient must not live, work, or sleep in a confined atmosphere. Much of the time should be should be spent out of doors, and large, well ventilated chambers, with open windows, should be used for sleeping apartments.

Special attention should be given to the skin. A warm or hot bath should be taken at least twice a week. The skin should be groomed daily, either after the bath or independent of it on the days on which he does not bathe. A tablespoonful or two of soda should be added to the bath.

Wool must be worn next to the body the year round, and the body at all times kept extra warm, as the temperature is constantly below normal, and chills are very liable to occur from slight causes. For the same reasons, cold and damp winds are to be avoided. Moderate exercise in the open air is very beneficial; it relieves the weary feelings, so common in the muscular system. Exercise should, however, never be carried to fatigue or exhaustion, as these often bring about a fatal complication (coma), even in the mildest cases. "Catching cold" is another danger to be carefully avoided at all times, as serious complications often arise therefrom.

The patient should drink freely of water—as freely as his inclinations prompt him.

